# Dietary Fish, Fish Nutrients, and Immune Function: A Review

**DOI:** 10.3389/fnut.2020.617652

**Published:** 2021-01-20

**Authors:** Carlos O. Mendivil

**Affiliations:** ^1^School of Medicine, Universidad de los Andes, Bogotá, Colombia; ^2^Section of Endocrinology, Department of Internal Medicine, Fundación Santa Fe de Bogotá, Bogotá, Colombia

**Keywords:** immunity, immune function, fish, omega-3, microbiota, seafood

## Abstract

Dietary habits have a major impact on the development and function of the immune system. This impact is mediated both by the intrinsic nutritional and biochemical qualities of the diet, and by its influence on the intestinal microbiota. Fish as a food is rich in compounds with immunoregulatory properties, among them omega-3 fatty acids, melatonin, tryptophan, taurine and polyamines. In addition, regular fish consumption favors the proliferation of beneficial members of the intestinal microbiota, like short-chain fatty acid-producing bacteria. By substituting arachidonic acid in the eicosanoid biosynthesis pathway, long-chain omega-3 fatty acids from fish change the type of prostaglandins, leukotrienes and thromboxanes being produced, resulting in anti-inflammatory properties. Further, they also are substrates for the production of specialized pro-resolving mediators (SPMs) (resolvins, protectins, and maresins), lipid compounds that constitute the physiological feedback signal to stop inflammation and give way to tissue reparation. Evidence from human observational and interventional studies shows that regular fish consumption is associated with reduced incidence of chronic inflammatory conditions like rheumatoid arthritis, and that continuous infusion of fish oil to tube-fed, critically ill patients may improve important outcomes in the ICU. There is also evidence from animal models showing that larger systemic concentrations of omega-3 fatty acids may counter the pathophysiological cascade that leads to psoriasis. The knowledge gained over the last few decades merits future exploration of the potential role of fish and its components in other conditions characterized by deregulated activation of immune cells and a cytokine storm like viral sepsis or COVID-19.

## Introduction

The immune system is responsible for allowing humans to survive in an environment replete with potentially pathogenic microorganisms, many of them living on or within our bodies. The influence of nutrition and metabolism on immune function is a topic of rising interest, to the point that the new discipline of “immunometabolism” has arisen in order to foster a deep comprehension of this crucial relationship (1). The impact of dietary intake on immune function derives not only from the direct physiological effects of its chemical constituents, but at least to a similar extent from the way it shapes the intestinal microbiota, which in turn is a primary stimulus and regulator of diverse immune cells (2). Fish as a food, as well as some of its chemical constituents have a series of properties that favorably modulate immune function. The objective of this article is to provide the reader with an overview of the evidence relating fish or fish constituents with immunity, including the main mechanisms involved and their translation into relevant observational and interventional evidence in humans. The review intentionally focuses on aspects not directly related to cardiovascular diseases.

## Nutrients With Immunomodulatory Properties Present in Fish

### Omega-3 Fatty Acids

Omega-3 polyunsaturated fatty acids (ω3-PUFA) are a type of fat found in very low concentrations in most terrestrial animals and plants, but particularly abundant in fish and seafood. Some of the fish with the highest content of omega-3 fatty acids are salmon (wild or farmed), herring, mackerel, sardines, tilefish, albacore tuna, pollock, halibut and trout (3, 4). There are multiple ω3-PUFA, but many of the immunoregulatory mechanisms that will be described in this paper are specific to the long-chain ω3-PUFAs eicosapentaenoic acid (EPA) (20:5, ω3) and docosahexaenoic acid (DHA) (22:6, ω3) or their derivatives. The human organism does not have the enzymatic repertoire required for the synthesis of ω3-PUFA in the amounts required under all physiological conditions, so they are considered conditionally essential nutrients. Even though there is a considerable amount of an 18-carbon ω3-PUFA (alpha-linolenic acid - ALA) in some plant oils, the efficiency of conversion of this ALA to EPA and DHA in the human organism is quite limited (5). Hence, fish and seafood may be a preferable dietary source of ω3-PUFAs.

**Figure 1:**
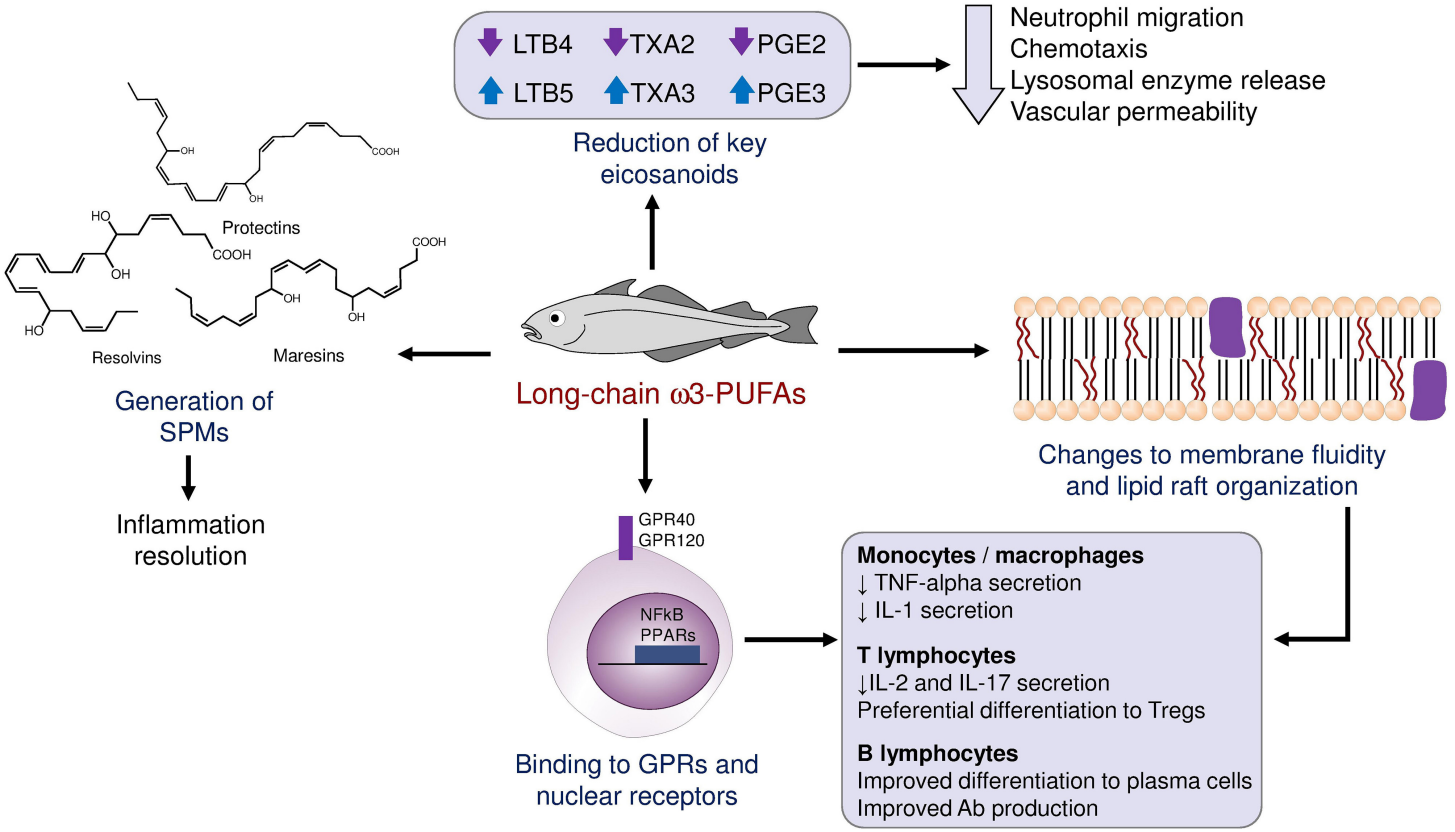
Schematic summary of the mechanisms through which ω3-PUFA influence the immune response and local inflammation.

ω3-PUFAs possess anti-inflammatory, vasodilatory, and antiaggregant properties. They induce these effects by a multitude of mechanisms that may be grouped as follows: i. Interference with the synthesis of chemical mediators normally derived from arachidonic acid (eicosanoids), ii. Generation of specialized pro-resolving mediators (SPMs), iii. Direct binding to cellular receptors, and iv. Changes to the fluidity and other physical properties of plasma membranes (Figure 1). Normally, arachidonic acid gives rise to prostaglandins of the “2” series (which induce fever, pain and inflammation), thromboxanes of the “2” series (which cause platelet activation and aggregation) and leukotrienes of the “4” series (which act as chemotactic molecules, attracting immune cells to the site of their release). When arachidonic acid is replaced by the ω3-PUFAs EPA and DHA, the final products are prostaglandins of the “3” series, which are only partially active; thromboxanes of the “3” series, which lack proaggregant activity; and leukotrienes of the “5” series, which have far weaker chemotactic activity than their series 4 counterparts (6).

#### The Role of Pro-resolving Mediators

An observation that for long time puzzled clinicians and researchers alike is that the normal inflammatory response tends to “resolve” over time after withdrawal of the initial insult, giving way to healing and reparation. A group of molecules collectively named “specialized pro-resolving mediators” (SPMs) has been identified as a critical player in this self-regulation of inflammation, including four families of compounds: Resolvins, protectins, maresins, and lipoxins. The first three of these mediators are biochemically derived from long-chain ω3-PUFAs and are largely responsible for preventing an indefinite prolongation of local immune responses (7). After a localized injury or trauma, the initial response is characterized by the production of PGE2 and prostaglandin I2 (PGI2), which bring about the cardinal inflammatory symptoms of redness (*rubor*), heat (*calor*), pain (*dolor*) and swelling (*tumor*). Simultaneously, leukotriene B4 (LTB4) attracts immune cells to the site of injury. Nonetheless, in presence of sufficient amounts of ω3-PUFAs, neutrophils and macrophages are able to synthesize SPMs from EPA and DHA, following separate biochemical pathways that were elucidated by analysis of exudates from resolving inflamed tissue in animal models (8). Resolvins 1 and 2 of both the “E” series (derived from EPA) and “D” series (derived from DHA) tend to be produced early during the resolution process, while resolvin D3 appears later on. Macrophages also produce maresin 1, which induces a phenotype switch from M1 (pro-inflammatory) to M2 (anti-inflammatory) in other macrophages. These SPMs also attract non-phlogistic macrophages that clean up cellular debris produced by the defense response (9). As this process proceeds, there is also a lipid mediator class-switching involving several autacoids, notably a switch from LTB4 to lipoxin 4, which prevents the extravasation and migration of blood neutrophils. The importance of these mediators in preventing a dysregulated and deleterious immune response as a consequence of systemic infection has been demonstrated in the context of bacterial sepsis (10), and influenza virus infection (11, 12).

Studies in human peripheral blood lymphocytes have shown that some SPMs, particularly resolvin D1, resolvin D2 and maresin 1, are able to reduce the secretion of cytokines by pro-inflammatory subsets of CD4+ T lymphocytes (Th1 and Th17), without negatively affecting their ability to proliferate (13). These same SPMs showed ability to bind to the fatty acid receptor GPR32, leading to a signaling cascade that prevented differentiation of naïve T-cells toward these inflammatory subtypes. In addition to regulating the proliferation and activation of pro-inflammatory T cell subtypes, an omega-3 rich diet has the ability to promote a Th2 immune response, characterized by the secretion of anti-inflammatory cytokines like IL-4 and IL-10 (14).

In diseases characterized by a deregulated, massive secretion of pro-inflammatory cytokines (the so-called cytokine storm), such as severe COVID-19, one of the key cytokines involved in pathogenesis is interferon-gamma (IFN-gamma) (15). Studies in mice models of acute infection have found that a high dietary intake of ω3-PUFAs significantly reduces IFN-gamma secretion (16). In fact, it has been hypothesized that some of the increased susceptibility of individuals with obesity to adverse outcomes after SARS-CoV-2 infection may be a consequence of their relative deficiency of SPMs derived from ω3-PUFAs (17). Furthermore, a recent proof-of-concept randomized trial in 100 patients with symptomatic COVID disease evaluated the effect of supplementation with icosapent ethyl, a highly purified preparation of EPA, on inflammation and symptomatology over 14 days. The active treatment group had 25% lower levels of C-reactive protein (CRP), signaling decreased systemic inflammation, and a significantly lower prevalence of symptoms assessed by the FLU-PRO scale (48 vs. 76%) (18). Interestingly, the reduction in plasma CRP correlated significantly with the improvement in the symptom scale. The effect of ω3-PUFAs on COVID-19 transmission among healthcare workers will be examined in a larger trial dubbed PREPARE-IT 1 (NCT04460651).

#### ω3-PUFAs, Membrane Properties and Immunity

Many of the effects of long-chain ω3-PUFAs on the functionality of immune cells are mediated at least in part by the impact of the properties of the cell membrane, especially the formation and confluence of lipid rafts. Lipid rafts are regions of the plasma membrane enriched in phospholipids and cholesterol, in which signaling proteins are usually concentrated (19). For example, a higher content of EPA and DHA in areas of the cell membrane prevents the recruitment into lipid rafts and subsequent dimerization of pattern-recognition receptors like TLR2 and TLR4 (20). Also, a greater concentration of ω3-PUFAs in the membrane phospholipids of antigen-presenting cells leads to biophysical changes that reduce their antigen presentation ability, and the subsequent activation of T lymphocytes (21). It may be said that long-chain ω3-PUFAs act as “traffic lights,” that direction immature T cells toward differing phenotypes and subclasses according to their concentrations at the moment of contact between the T cell and other key stimuli. EPA, DHA and their pro-resolving metabolites have the ability to influence the trans-endothelial migration and motility of memory T-cell subsets (22). In general, ω3-PUFAs and SPMs reduce the trafficking and retention of T cells to inflamed tissues.

The consequences of a larger exposure to, and plasma membrane contents of ω3-PUFAs, seem to be different for B lymphocytes. Several studies in animal models have shown that ω3-PUFAs foment the migration of mature B lymphocytes out of the spleen, their homing into tissues where an immune response is taking place, and their differentiation to antibody-producing plasma cells (21).

Studies in mice infected with pH1N1 influenza virus have found that 17-hydroxy DHA, a SPM derived from DHA, is able to induce in B cells the expression of the transcription factor Blimp-1, a master regulator of their differentiation to antibody-secreting plasma cells (23). The impact of ω3-PUFAs on B cell function has been particularly well-documented in the context of obesity. It is well-known that obese humans and animals display impaired B cell responses in terms of differentiation and antibody production when exposed to a pathogen or vaccine (24). Interestingly, obese mice display reduced production of ω3-PUFAs-derived SPMs at immunologically relevant tissues like the spleen (25). Mice who ingest a western-type diet show lower antibody titers than controls after infection with an influenza A virus, while mice whose diet was supplemented with DHA display higher titers. This difference could not be explained as a direct effect of DHA on B cells, but rather as a consequence of enhanced production of SPMs derived from DHA, like 14-hydroxydocosahexaenoic acid, 17-hydroxydocosahexaenoic acid and protectin DX (24). In a pilot trial in humans with obesity, dietary supplementation with 4 g/day of fish oil for 12 weeks induced a reduction in the number of circulating memory and plasma B cells, with an attenuation of their production of IgM, but no effect on their IgG secretion post-stimulation *ex-vivo* (26). In this respect, not all ω3-PUFAs seem to be equal. An interesting mechanistic study compared the effect of a 5-week diet supplemented with DHA-enriched or EPA-enriched fish oil on the *ex-vivo* functionality of mice B lymphocytes (27). Surprisingly, DHA resulted in less clustering of membrane microdomains, while EPA showed caused increased clustering, a difference that was accompanied by disparate effects on the expression of cytokine genes. *In vitro* studies of neutrophil adhesion and degranulation have shown that contact with ω3-PUFAs facilitate and optimize the release of their granules (28, 29).

#### ω3-PUFAs, Cellular Receptors and Immunity

Long-chain ω3-PUFAs are directly recognized by membrane receptors like G protein-coupled receptors GPR120 (type 4 fatty acid receptor) (30) or GPR40, and by several nuclear receptors that act as transcription factors in macrophages. Binding of ω3-PUFAs to GPR120 regulates the synthesis and release of key cytokines like tumor necrosis factor-alpha (TNF-alpha), interleukin 1 (IL-1) and prostaglandin E2 (PGE2), upon exposure to bacterial components. On the other hand, ω3-PUFAs may directly bind nuclear receptors and elicit a concerted transcriptional response in immune cells. One family of such nuclear receptors is that of PPARs (peroxisome-proliferator activated receptors). Both DHA and EPA are able to bind PPAR-gamma and dose-dependently reduce the percentage of cytokine-expressing Th cells when incubated with primary mononuclear cells from normal individuals (31). Another transcription factor with a key role in orchestrating the inflammatory response is nuclear factor kappa-B (NFkB). Cellular exposure to ω3-PUFAs results in phosphorylation of IkB, a protein that binds to and inactivates NF-kB. As a consequence, NFkB may not enter the nucleus and the whole downstream pro-inflammatory signaling cascade is not activated (32).

Studies of the influence of lipids on lymphocyte function have revealed that exposure to ω3-PUFAs has a direct effect on the maturation of T lymphocyte precursors, preventing their differentiation toward the most pro-inflammatory subtypes, Th1 and Th17 (8). This effect has been well-documented in mouse models of contact and atopic dermatitis, in which dietary supplementation with long-chain ω3-PUFAs led to reduced Th1 and Th2 responses, and to a large improvement in the macroscopic signs of dermatitis severity (33). ω3-PUFAs also impact directly on the maturation and antigen-presenting capacity of dendritic cells, an effect probably mediated by reduced NFkB activation (34).

In sum, ω3-PUFAs impinge on the immune response through multiple, many times redundant mechanisms, and long-chain ω3-PUFAs from fish may play a role in conditions characterized by a deregulated immune response.

### Melatonin

Melatonin, or N-acetyl, 1-5-methoxytryptamine, is a derivative of the essential aminoacid tryptophan generated through the acetylation and methylation of serotonin. The most extensively studied function of melatonin is the regulation of the sleep-wake cycles in concert with serotonin in the pineal gland, but melatonin also plays an important role in the modulation of immune function. Two G-protein coupled receptors for melatonin have been identified (MT1 and MT2), primarily expressed in the brain but also present in the thymus and spleen (35). A wide variety of immune cells express melatonin receptors and respond to its local concentrations, including B cells, CD4 and CD8 T cells, monocytes, neutrophils and NK cells (35–37). In general, stimulation of immune cells by melatonin results in a greater proliferative capacity and a modification in the pattern of cytokines released (38). Melatonin promotes differentiation of T cell precursors toward the regulatory T cell (Treg) phenotype, and activates existing Tregs to produce IL-10 (39). Conversely, melatonin suppresses differentiation of Th17 cells and hence reduces IL-17 secretion (40). Studies in synovial fibroblasts have found that melatonin induces a reduction in the secretion of IL-1 and TNF-alpha, an observation with potential relevance for the pathogenesis of inflammatory joint diseases (41). A study in zebrafish found that exogenous melatonin administration was able to inhibit migration of neutrophils to a local inflammation site, an effect mediated by inhibition of signaling through the extracellular signal-regulated kinases (ERK) pathway (39). Similarly, studies of leukocyte migration through the peritoneal epithelium of mice have found that melatonin prevents the expression of the adhesion molecule CD18 on granulocytes, an effect mediated by the MT2 receptor (42). A very interesting observation in human primary peripheral mononuclear cells is that melatonin may exert an immunomodulatory and anti-inflammatory effect in cells from patients with systemic lupus erythematosus, but such effect is entirely different in cells from normal individuals (43).

Within the context of autoimmune diseases, the link between seasonal variation in melatonin levels (associated with night length) and severity among patients with multiple sclerosis was recently explored in a very comprehensive study (44). Circulating melatonin levels correlated negatively with disease activity in humans, while exogenous administration of melatonin alleviated disease severity in a mouse model of multiple sclerosis. Melatonin blocked the differentiation of pathogenic Th17 cells through induction of the repressor transcription factor Nfil3, and increased formation of Treg cells via activation of the ERK pathway and transactivation of the IL-10 promoter. These results confirmed and expanded prior observations in the autoimmune encephalomyelitis (EAE) mouse model of multiple sclerosis (45).

Thus, melatonin has a relevant involvement as a regulator of the immune response. Unfortunately, melatonin is relatively scarce in the diet. Dietary fish is an excellent source of melatonin, providing about 3.7 ng per gram of raw food, or approximately 300–350 ng per serving (36).

## Fish Consumption, Intestinal Microbiota and Immune Function

The human gut houses an enormous number of microorganisms including protozoans, viruses and fungi, but specially bacteria, with up to 1 billion bacteria per gram of intestinal content (46). This community of microorganisms in which more than 400 different species are represented is referred to as the intestinal microbiota. Bacteria benefit from the stable, nutrient-rich environment provided by the foods we ingest, while providing us vitamins like cobalamin and vitamin K, enhanced digestion of certain foods, and beneficial short-chain fatty acids. Naturally, the relative abundance of each bacterial species is strongly influenced by the diet. Moreover, bacteria from the normal microbiota compete with, and inhibit the growth of, potentially pathogenic microorganisms (46).

More than 90% of the normal intestinal microbiota is composed of organisms from the phyla *firmicutes, bacteroidetes, actinobacteria, proteobacteria*, and *verrucomicrobia*. With human aging, characteristic changes occur in the intestinal microbiota including a reduction of *bacteroidetes* (like *bacteroides*) and *actinobacteria* (like *bifidobacterium*), and a concomitant increase in potentially pathogenic *firmicutes* (like *clostridium*).

Mechanistic studies have demonstrated that dietary lipids have a strong influence on the composition of the intestinal microbiota and its impact on the immune system. Compared with mice fed a lard-rich diet, mice who consumed a fish oil-rich diet for 11 weeks displayed reduced inflammation in adipose tissue and a markedly different signature in their microbiota, characterized by greater abundance of *Akkermansia* and *Lactobacillus*, and reduced *Bacteroides* in their cecum. Actually, an estimated 24% of the variability in the composition of the intestinal microbiota could be attributed to dietary fat type. The fish oil diet also induced lower levels of circulating pro-inflammatory bacterial sub-products (47). The large influence that ω3-PUFAs may have on the intestinal microbiota is very well-illustrated by the fact that fat-1 transgenic mice, which may constitutively produce ω3-PUFAs, have a healthier microbiota, a better intestinal barrier function and consequently lower levels of circulating endotoxin. Moreover, transplantation of fecal microbiota from fat-1 mice to their wildtype littermates corrected many of the intestinal, immunological and metabolic alterations induced by an obesogenic, western-type diet (48).

Interventional studies in both healthy children (49) and in adults with type 2 diabetes (50) have proven that the regular ingestion of ω3-PUFAs is accompanied by an increase in the proportion of *bacteroidetes* and other beneficial butyrate-producing bacteria. Dietary ω3-PUFAs also reduce the proportion of harmful bacteria from the *clostridia* class such as *coprococcus* and *faecalibacterium* (51). Several products generated by bacterial metabolism help mature and regulate the mucosal immune system, among them short-chain fatty acids, the aminoacids tryptophan, taurine and arginine, and several other polyamines. The dietary ingestion of fish favors the proliferation of normal commensal bacteria, increasing production of all these compounds by the intestinal microbiota (28).

Dietary ω3-PUFAs also favor the functional integrity of the mucosal barrier through various mechanisms. On the one hand, they directly hamper the adherence and subsequent colonization of the mucosal wall by some bacteria (52). On the other hand, they induce cells of the mucosal epithelium to express higher levels of the protein zonulin, resulting in tighter inter-cellular unions and prevention of mucosal colonization through the paracellular pathway (53).

There is also evidence about a role of the intestinal microbiota in the maturation of the immune system at the systemic level. As expected, mice kept under sterile conditions produce significantly less immune cells in their intestinal wall, produce much less secreted antibodies (immunoglobulin A - IgA) and harbor less CD4+ lymphocytes in their intestinal lymphoid tissue. But in addition to this, the lack of exposure to the intestinal microbiota also leads to underdeveloped spleen ad lymph nodes, and to markedly reduced concentrations of total circulating antibodies (46). Even though humans are born with a nearly sterile intestine, early exposure to the intestinal microbiota promotes the differentiation of regulatory lymphocyte subpopulations, many of which secrete interleukin 10 (IL-10) and modulate the response to new antigenic challenges, preventing the development of allergies and autoimmune diseases (54).

One of the species of intestinal bacteria with most positive effects on human health is *Akkermansia muciniphila*, a gram-negative bacterium that lives in and feeds from the mucus produced by the intestinal mucosa. The metabolism of *Akkermansia* generates the short-chain fatty acids acetate, propionate and butyrate, which promote integrity of the mucosal barrier and protect it from invasive species (55). *Akkermansia* also produces antibiotic peptides that suppress other potentially pathogenic bacteria (56), and stimulate regulatory lymphocytes (57). Dietary ω3-PUFAs stimulate the proliferation of *Akkermansia* (58). In a population study of 876 adult women, plasma concentrations of DHA and the frequency of dietary fish consumption correlated significantly with the abundance of butyrate-producing bacteria in the gut of study participants (59). However, not all studies of ω3-PUFAs supplementation have reported produced equally beneficial effects on the intestinal microbiome; for example, the effect of ω3-PUFAs on the abundance of bacteria from the phylum *Proteobacteria* have been contradictory, with some studies finding relative increases and other relative decreases (60).

## Fish Consumption and Inflammatory Disorders

Early studies of native populations from Greenland, whose diet was based almost exclusively on ω3-PUFAs-rich fish and seafood, revealed extremely low rates of chronic inflammatory diseases like atherosclerosis and psoriasis (61). These findings highlighted that in many morbid conditions, the largest degree of damage is imparted not by a foreign invader, but by a deregulated immune response that ends up harming the very organism it is trying to defend. This pattern has been observed not only for chronic diseases but also in the context of septicemia, influenza virus infection and in the severe infection by SARS CoV-2 (62). Given the multitude of mechanisms by which long-chain ω3-PUFAs and their derivatives modulate the immune response, there is a physiologic rationale for their potential use in the prevention or treatment of diseases characterized by chronic systemic inflammation or acute deregulated cytokine secretion.

A recent meta-analysis synthesized the available evidence about the effect of the ingestion of ω3-PUFAs on plasma concentrations of inflammatory mediators, the study included a total of 18 trials with 826 individuals (63). ω3-PUFAs significantly reduced circulating levels of thromboxane B2 (TXB2) and LTB4, indicating a lower degree of platelet activation and lower production of chemotactic molecules. Other studies have focused on the effect of ω3-PUFAs on activation of immune cells. When macrophages stimulated with bacterial lipopolysaccharide were simultaneously incubated with physiologically relevant concentrations of ω3-PUFAs, their production of the proinflammatory cytokines IL-1 beta, IL-6 and TNF-alpha was reduced by more than half (64). This effect has been replicated in adipose tissue, which has emerged as a major source of cytokines in multiple metabolic diseases (65).

The impact of dietary or supplemental ω3-PUFAs on rheumatoid arthritis (RA), the epitome of chronic inflammatory diseases, has been extensively assessed. Results from seven observational studies exploring the association between fish consumption and risk of RA were compiled in a meta-analysis (66), revealing that consumers of 1–3 servings of fish per week had a 24% lower risk (RR = 0.76, 95% CI: 0.57–1.02) compared to non-consumers. However, a large observational study of US women published after this meta-analysis (67) did not find a significant association between fish intake ≥4 servings/week and risk of new-onset seropositive or seronegative rheumatoid arthritis. The only indication of benefit was among the subgroup of women <55 years old, in whom the risk for seronegative RA was 45% lower for women with frequent fish intake. High fish intake also seemed to attenuate the increased risk of RA among younger women who smoked.

Concerning patients already diagnosed with RA, a cross-sectional study found a lower severity of symptoms according to the DAS28-CRP (Disease Activity Score in 28 joints using the C-reactive protein) score among subjects consuming fish ≥2 times/week (68). In a clinical trial that included 49 patients with rheumatoid arthritis (69), ω3-PUFAs supplementation at 80 mg/Kg/day for 36 weeks reduced the number of painful joints by 34% and the number of inflamed joints by 12%. These improvements were accompanied by a 19% decrease in plasma LTB4 and a 54.7% decrease in plasma IL-1. Moreover, patients with diagnosed rheumatoid arthritis who ingested a high-dose ω3-PUFAs supplement for 1 year had lower rates of therapeutic failure and required lower doses of the standard anti-rheumatic drugs (70). Results from 20 small clinical trials of ω3-PUFAs in patients with pre-existing RA were compiled in a recent meta-analysis (71). Dietary administration of ω3-PUFAs improved eight markers of disease activity and led to significant decreases in circulating LTB4. However, the quality of the trials was generally low, so these results must be interpreted with caution.

Chronic deranged inflammation is also a key factor in inflammatory intestinal diseases (Crohn's disease and ulcerative colitis). A study in a rat model of Crohn's disease (2,4,6-trinitrobenzene sulfonic acid [TNBS] exposure) evaluated the effect of the intragastric infusion of 20 mg/Kg/day of ω3-PUFAs for 60 days on multiple aspects of disease severity and progression. ω3-PUFAs markedly attenuated histologic hallmarks of colonic inflammation, while increasing mucosal expression of PPAR-gamma and reducing the expression of the mRNAs for the pro-inflammatory transcription factor NFAT and for IL-2 (72). Psoriasis is yet another example of autoimmune chronic inflammatory disease. A key player in the pathogenesis of psoriasis is the pro-inflammatory cytokine IL-17. Imiquimod-treated mice (an animal model of psoriasis) who were genetically programmed to generate endogenous ω3-PUFAs, had significantly lower IL-17 production and reduced inflammatory activity relative to their wild-type counterparts (73).

An interesting small, proof-of-concept clinical trial assessed the effect of supplementing the diet of Thai schoolchildren aged 9–12 with fish oil (2 g/day, containing 200 mg EPA and 1,000 mg of DHA), using with 2 g of soybean oil as control, over 6 months (74). Despite no significant difference in plasma levels of pro-inflammatory cytokines between groups, children in the fish oil group experienced significantly fewer and shorter episodes of acute diseases, mostly upper respiratory infections.

In summary, long-chain ω3-PUFAs have shown consistent immunomodulatory effects *in vitro*, in animal models of chronic disease and in small clinical trials of humans, particularly in RA. However, their incorporation into routine clinical practice would require larger, well-designed randomized trials.

## Fish Oil In Critically Ill Patients

Many patients with acute, severe diseases typically managed at the intensive care unit suffer from a state of massive, generalized and disorganized inflammation denominated “immunoparalysis” (75). As ω3-PUFAs regulate the immune response, their ability to prevent adverse outcomes in such patients has been the subject of much interest. In fact, the accumulation of convincing evidence has led to the recommendation to use fish oil in the enteral formulas for patients with acute respiratory distress syndrome (ARDS) in several guidelines (76). ARDS has recently gained particular attention, as it is one of the usual manifestations of severe forms of COVID-19 (77). In a study of ICU patients with sepsis under mechanical ventilation in Spain, the addition of ω3-PUFAs and antioxidants to their enteral formula resulted in a shortening of their ICU stay by an average of 2 days (78). The INTERSEPT study evaluated the effect of the continuous infusion for 7 days via tube-feeding of two isocaloric and nitrogen-matched diets containing or not a lipid mixture with EPA, gamma-linolenic acid and antioxidants, in patients with sepsis (79). Patients in the lipid-enhanced diet developed less severe sepsis, required less mechanical ventilation and had shorter ICU stays. Surprisingly, however, no significant effect was observed on total mortality. The results from INTERSEPT cannot be attributed solely to the EPA component of the enteral nutrition formula, but taken in conjunction with biological plausibility and prior evidence, they do suggest a positive impact of ω3-PUFAs on critically ill patients.

Results from these and other studies were consolidated in a meta-analysis that concluded that continuous infusion of ω3-PUFAs to patients in the ICU resulted in mean reductions of 32% in mortality, 3.6 days in ICU stay and 4.8 days in duration of mechanical ventilation (75). It is important to note, however, that these benefits were only present for studies in which ω3-PUFAs-containing formulas were given as a continuous infusion, while bolus administration has shown no beneficial effect and may actually induce a prolongation the longer ICU stay, more requirement of mechanical ventilation and a greater risk of diarrhea (80, 81). The difference seems to reside in the induction of sustained, higher levels of ω3-PUFAs in circulation, which may only be attained with continuous infusion (82). Also, the very high load of fat in a bolus of ω3-PUFAs-enriched formula (up to 120 mL) may induce malabsorption and subsequent diarrhea.

## Discussion

Several lines of evidence from *in vitro*, translational, epidemiological and clinical evidence suggest a positive impact of fish and marine ω3-PUFAs on immune function. The mechanisms implicated are still not fully understood but likely involve both direct effects of their chemical constituents and indirect effects mediated by changes in the intestinal microbiota and its metabolic byproducts (postbiotics). ω3-PUFAs serve as surrogate substrates for arachidonic acid, leading to the production of a different repertoire of eicosanoid mediators and resulting in anti-inflammatory, vasodilating and antiaggregant effects. They also serve as substrates for the synthesis of pro-resolving lipids like resolvins, protectins, and maresins all of which orchestrate the physiological resolution of inflammation and the transit to reparation and healing. ω3-PUFAs also regulate the immune response through binding to membrane and nuclear receptors, and through the induction of biophysical changes in the plasma membrane. The immunoregulatory attributes of fish and fish oil are reflected in the negative association between dietary fish intake and risk of chronic inflammatory diseases. Future research should explore the generalizability of these findings in sufficiently powered clinical trials, not only in chronic inflammatory diseases but also in other diseases characterized by immune dysfunction such as allergy or COVID-19.

## Author Contributions

CM conceived the review, executed the search and evaluation of the collected evidence, wrote, and reviewed the final manuscript.

## Conflict of Interest

The author declares that the research was conducted in the absence of any commercial or financial relationships that could be construed as a potential conflict of interest.
