# Corrigendum: Dietary Fish, Fish Nutrients, and Immune Function: A Review

**DOI:** 10.3389/fnut.2021.693773

**Published:** 2021-05-19

**Authors:** Carlos O. Mendivil

**Affiliations:** ^1^School of Medicine, Universidad de los Andes, Bogotá, Colombia; ^2^Section of Endocrinology, Department of Internal Medicine, Fundación Santa Fe de Bogotá, Bogotá, Colombia

**Keywords:** immunity, immune function, fish, omega-3, microbiota, seafood

A correction has been made to **Nutrients with immunomodulatory properties present in fish, Melatonin**, third paragraph.

The sentence in mention has been changed from “3.7 mg per gram of raw food, or approximately 300–350 mg”, to “3.7 ng per gram of raw food, or approximately 300–350 ng.”

The author apologizes for this error and states that this does not change the scientific conclusions of the article in any way. The original article has been updated.

